# A preliminary microscopic and molecular epidemiological survey of endoparasites within wild-caught and UK captive-bred reptiles: Assessing a potential parasitic disease public health risk?

**DOI:** 10.1016/j.ijppaw.2025.101039

**Published:** 2025-01-11

**Authors:** Shea Murray, Lucas J. Cunningham, Paul Rowley, Edouard Crittenden, Nicholas R. Casewell, E. James LaCourse, J. Russell Stothard, Alexandra Juhász

**Affiliations:** a Department of Tropical Disease Biology, Liverpool School of Tropical Medicine, Liverpool, L3 5QA, UK; b Centre for Snakebite Research & Interventions, Liverpool School of Tropical Medicine, Liverpool, L3 5QA, UK; c Institute of Medical Microbiology, Semmelweis University, H-1089, Budapest, Hungary

**Keywords:** Endoparasites, *Giardia*, Herpetology, *Ophidascaris*, Pentastomes, Reptiles, *Strongyloides*

## Abstract

In the UK, exotic reptiles are increasingly popular as pets, and housed in zoological collections, whilst venomous snakes of medical importance have long been the focus of herpetological studies. As all reptiles can harbour protist and helminth parasites, some of these may carry tangible zoonotic risk. This study utilised traditional and molecular diagnostic techniques, including sedimentation-flotation, real-time polymerase chain reaction (rtPCR), and necropsy, to investigate endoparasite infections in captive-bred (CB) and wild-caught (WC) reptiles. Representative animals originated from pet shops, zoological and private collections as well as those housed in research herpetariums. Parasitic infections were detected in 21.1% (n = 109) of samples from 58 reptile species across 12 families. The most prevalent infections included nematodes (17.4%), cestodes (0.9%) and protists (3.7%). The nematodes, particularly strongylid (9.3%) and ascarid (5.6%) species, being the most common. Of particular interest, zoonotic genera, *Ophidascaris* and *Giardia* were identified. When possible, necropsy revealed latent infections, including prepatent stages of the hookworm *Kalicephalus* sp. and pentastomid larvae in *Echis ocellatus* snakes. These accounted for 55.6% of all parasitic infections. Real-time-PCR methods detected additional co-infection overlooked by microscopy, whilst necropsy provided additional insights. These findings highlight the need in the UK for better parasitic screening protocols to enhance captive reptile welfare, mitigate zoonotic risks and safeguard public health.

## Highlights

• 21.1% prevalence of endoparasites detected in captive and wild reptiles.
• 55.6% of necropsied reptiles showed latent parasitic infections.
• Molecular methods revealed hidden infections missed by microscopy.
• Findings emphasize the need for better biosecurity in reptile management.
• Comprehensive diagnostics improve reptile welfare and mitigate zoonotic risks.

## Graphical abstract

**Figure:**
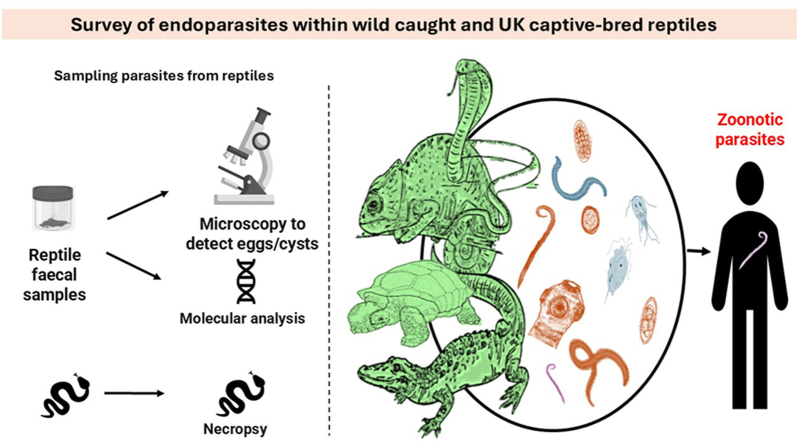

## 1 Introduction

The increasing integration of snakes and other reptiles into captive or research settings, driven by conservation, the exotic pet trade, and medical research, has heightened concerns about underlying parasitic infections which may have zoonotic potential (Ellerd et al., 2022; Guardone et al., 2024; Sazmand et al., 2024). Endoparasites, such as protists, and helminths can severely affect reptile health, leading to gastrointestinal disorders, weight loss, anaemia, poor condition and sometimes death (Jacobson and Garner, 2020). Both captive-bred (CB) and wild-caught (WC) reptiles are susceptible to these infections, and certain parasites are known zoonotic risks to their handlers, keepers and owners (Kelehear et al., 2013; Hossain et al., 2023). Parasitic genera such as *Giardia* and *Ophidascaris* are notably associated with zoonotic transmission (He et al., 2024; Hossain et al., 2023). Furthermore, many reptiles can act as asymptomatic carriers of various parasites, presenting a cryptic infection risk to humans despite appearing healthy. This zoonotic potential of human-reptile interface underscores the relevance of parasitic infections in both veterinary medicine and public health (Mendoza-Roldan et al., 2020; Leung, 2024).

Although studies have examined parasitic infections in reptiles, most focus on single species or use limited diagnostic techniques, often neglecting molecular methods or comparisons between captive-bred and wild-caught reptiles (Rataj et al., 2011; Wolf et al., 2014; Hallinger et al., 2020; Bogan et al., 2024; Zhang et al., 2024). Given the zoonotic potential and welfare concerns associated with parasitic infections, there is a clear need for comprehensive screening of parasites in reptiles across both captive and wild environments, particularly exotics transported to, and/or maintained in the UK, such as those destined for/within pet shops, private and public zoological collections, as well as in research herpetatriums. This study aims to address these gaps by combining traditional and molecular methods to evaluate parasitic infections in CB and WC reptiles, with a focus on zoonotic implications and management strategies.

## 2 Methods

### 2.1 Sample collection and microscopical identification of parasites

From December 2023 through June 2024, coprological analyses were performed on 109 donated, reptile faecal samples. To achieve a comprehensive spectrum of endoparasites, efforts were made to collect samples from a diverse range of reptile (boid, colubrid, elapid, pythonid and viperid snakes, chameleons, crocodiles, alligators and caimans, geckos, iguanas, monitor lizards and tortoises) species, incorporating both CB and WC individuals from a research herpetarium (n = 24), zoological institutions (n = 56), pet shops (n = 15) and private collectors (n = 14) (Online Supplement). All samples were stored at 4 °C until analysis. Selection criteria included the availability of an adequate quantity of faecal material (minimum 6 g) and the condition of the samples, which should not be grossly contaminated with sand or soil. Approximately 1 g aliquots of each sample were frozen for molecular testing. Participating herpetaria, institutes, pet shops and owners were requested to provide information with details for each animal, including signalment (species, sex, age), husbandry conditions (such as the duration of ownership), previous parasitological examinations, and any anthelminthic treatments (Hallinger et al., 2018) (Online supplement).

Faecal samples were processed using the combination of sedimentation, filtration, and flotation techniques to concentrate parasitic eggs, cysts, and larvae, following established parasitological protocols (Faust et al., 1939; Hoffman et al., 1934; Zhang et al., 2024). Approximately 4 g of faecal material was soaked in 100 ml of distilled water for 10 min to loosen the sample and improve the separation of parasites from debris. Samples containing dry or desiccated material were soaked longer to ensure proper rehydration. After soaking, each sample was filtered through a series of sieves with decreasing meshpore diameters (425 μm and 212 μm) to remove large debris, including hair and undigested material. The filtrate was collected in a tray and then passed through a Flukefinder apparatus (Richard Dixon, Soda Springs, ID, USA), which contained two further fine mesh filters. The first filter was examined for adults and large parasite stages by dissecting microscope, while the second filter was backflushed with water to concentrate the remaining material. Following filtration, on the second filter, the retained sample was transferred to a 15 mL centrifuge tube and spun at 2000 rpm for 2 min. The supernatant was carefully decanted, and 5 ml of zinc sulphate solution (specific gravity: 1300 g/l) was added to the sediment. The mixture was vigorously shaken to re-suspend the sediment, followed by a second centrifugation. The zinc sulphate flotation method was chosen to enrich and identify parasites. Particles on the surface of the solution were picked up with a glass rod, smeared across the surface of a slide and examined for recognisable parasites by compound microscope following previously described protocols (Navone et al., 2005). Cestode, nematode and protozoa species were identified morphologically under a microscope based on prior descriptions (Barnard and Upton, 1994; Schneller et al., 2008 ; Hedman and Rådström, 2013; Beck and Pantchev, 2013).

### 2.2 Necropsy

Necropsy was performed on nine snakes across 6 species, with carcasses obtained from a research herpetarium (Table 1., Online supplement). The animals died of natural causes due to factors such as traveling stress, diseases or age. No animals were euthanised for the purposes of this study.

**Table 1:** Performed necropsies of reptiles, reptile family, and origin (n = 8) regarding infection rate with endoparasites (%).

Reptile family (No. of different examined species)	No. examined	Origin (WB/CB)	Positive for endoparasites (%)
*Elapidae* (2)	2	WB (2)	1 (50)
*Vipeidae* (7)	7	WB (5)/CB(1)/n.a. (1)	4 (57.1)

The body cavity was opened with a midline incision, starting at the cloaca and extending anteriorly. Organs such as the lungs, stomach, intestines, kidney and liver were inspected macroscopically for visible parasites, cysts, or lesions. The necropsy procedures involved a detailed examination of the gastrointestinal tract and other internal organs to identify any parasitic infections (Terrell and Stacy, 2007). In addition to these necropsies, anamnestic data were collected from each dissected snake (Online supplement).

Samples of any abnormal tissues, parasites, or cysts were collected for further microscopic and molecular analysis. Parasites were identified morphologically and were stored in 70% ethanol for molecular work. Afterwards, intestinal contents were collected, and examined through sedimentation-flotation analysis and rtPCR. Morphological and molecular confirmation of parasitic infections was conducted using the methods described above.

### 2.3 DNA extraction and real-time (rt)PCR

Total DNA was extracted from the faecal samples to allow for the molecular identification of parasitic infections. Initially, 0.1 g of each faecal sample was weighed out and added to a Magna Lyser tube containing 0.9 g of 1.4 mm ceramic beads to facilitate mechanical disruption. Samples containing hair or undigested matter were cut into smaller fragments primarily to aid in sample disruption, as the hair was not removed and any potential inhibitors, such as melanin, would still be present (da Silva Argôlo et al., 2013).

After the addition of 251 μl of a 2% Polyvinylpolypyrrolidone (PVPP)/Phosphate-buffered saline (PBS) suspension, samples were vortexed for 10 s and frozen at −70 °C for 30 min to improve DNA extraction efficiency. After thawing at room temperature, samples were subjected to mechanical disruption by bead-beating for 30 s at 3000 rpm using the Magna Lyser (Roche Diagnostics, Germany). Following bead-beating, the samples were centrifuged at 8000 rpm for 30 s, in line with standard faecal DNA extraction (van den Berg et al., 2006).

Each sample were processed using the QIAamp DNA Mini Kit (QIAGEN, Hilden, Germany) manufacturer's protocol (Verweij et al., 2007). The purified DNA was eluted by adding 100 μl of preheated AE buffer (55 °C) to the spin column and centrifuging at 8000 rpm for 1 min. DNA concentrations were measured using a NanoDrop spectrophotometer (Thermo Scientific, USA), and samples were stored at 4 °C until rtPCR analysis.

### 2.4 Semi-quantitative rtPCR

The rtPCR was performed with genus specific probes/primers to detect *Giardia*, *Strongyloides*, *Trichomonas*, and *Ophidascaris* infections in three different reactions. ‘*Reaction 1*’ consisted of a triplex reaction targeting *Giardia* and *Strongyloides* using genus specific primers and Taqman probes with additional primers and probe for the phocine herpes virus (PhHV) internal positive control. ‘*Reactions 2 and 3*’ consisted of singleplex evagreen-rtPCR targeting *Trichomonas* and *Ophidascaris* respectively.

The primers and probes used in ‘*Reactions 1–3*’ are listed in Table 2 and were adapted from existing primers and probes described in the literature (Cepicka et al., 2005; van den Berg et al., 2006; Verweij et al., 2010; Jothikumar et al., 2021). The primer concentrations used in ‘*Reactions 1–3*’ were as follows: 250 nM (*Giardia*), 200 nM (*Strongyloides*), 100 nM (PhHV), 100 nM (*Trichomonas*) and 600 nM (*Ophidascaris*). The concentration of probes for ‘*Reaction 1*’ was 100 nM for all three targets (*Giardia, Strongyloides* and PhHV). Therefore, each sample was subjected to three reactions, with the first being a multiplex probe-based reaction targeting *Giardia* sp., *Strongyloides* sp. and the internal PhHV control. ‘*Reactions 2 and 3*’ were singleplex EvaGreen® rtPCRs targeting *Trichomonas* sp. and *Ophidascaris* sp. respectively.

**Table 2:** rtPCR ‘*Reactions 1–3*’, listing primers, probes and their targets.

Reaction	Target group	Target gene	Primer name	Primer sequence (5′-3′)	Reference
1	*Giardia* spp.	18S RNA	18SJVGF	ATCCGGTCGATCCTGCCG	Jothikumar et al. (2021)
			18SJVGR	ACGTCTTGGCGCCGGGTT	
			Probe 18SJVGP	[FAM]CGGCGGACGGCTCAGGA[BHQ1]	
	*Strongyloides* spp.	18S RNA	1530F	GAATTCCAAGTAAACGTAAGTCATTAGC	Verweij et al. (2010)
			1630R	TGCCTCTGGATATTGCTCAGTTC	
			Probe 1586T	[HEX]ACACACCGGCCGTCGCTGC[BHQ1]	
	PhHV-1	gB	267s	GGGCGAATCACAGATTGAATC	van den Berg et al. (2006)
			337as	GCGGTTCCAAACGTACCAA	
			Probe 305tq	[Cy5]TTTTTATGTGTCCGCCACCATCTGGATC[BHQ3]	
2	*Trichomona* spp.	16S rDNA	16SL	TACTTGGTTGATCCTGCC	Cepicka et al. (2005)
			16SR1	TCACCTACCGTTACCTTG	
3	*Ophidascaris* spp.	ITS 1, 5.8S RNA	NC13F	ATCGATGAAGAACGCAGC	This paper
			NC2R	TTAGTTTCTTTTCCTCCGCT	

Reaction mixes totalled 12 μL consisting of 2 μl of DNA template, n μL of previously mentioned primer concentration, 6 μL supermix, either AppProbe No ROX Mix (‘*Reaction 1*’) or Type-it HRM mix (‘R*eactions 2–3*’), with the remaining volume being made up of nuclease-free water.

Cycling conditions followed the recommendations of the manufacturer for each supermix with the following annealing temperatures being used for ‘*Reaction 1, 2 and 3*’, respectively: 60 °C, 50 °C and 55 °C. All reactions were run for 40 cycles with negative controls and positive controls for the following: *Giardia, Strongyloides* and *Trichomonas*.

## 3 Results

### 3.1 Endoparasites prevalence and diversity in faecal samples

Endoparasitic infections were detected in 21.1% (23/109; 95% CI: 13.4%–28.8%) of faecal samples, identified through sedimentation-flotation and rtPCR methods (Table 3, Table 4). Infections included the helminths, nematodes (17.4%), protists (3.7%), cestodes (0.9%), and pentastomes, distributed across reptile hosts (Table 5). Among nematodes, ascarid and strongylid species were most prevalent, comprising 9.3% and 5.6% of infections, respectively.

**Table 3:** Examined faecal samples of reptile and origin of sender (total n = 109) regarding infection rate with endoparasites (%) (n.a.Fstl: not applicable).

Reptile family (No. of different examined species)	No. examined	Origin (No. from research herp./pet shops/zoo/private)	No. of Wild (WB) or Captive (CB) bread	Positive for endoparasites (%)
*Aligatoridae* (7)	14	0/0/14/0	n.a.	0
*Boidae* (2)	3	0/1/0/1	CB (3)	1 (33.3)
*Chamaeleonidae* (1)	3	0/0/3/0	n.a.	2 (66.7)
*Colubridae* (8)	13	0/0/5/7	WB(1)/CB(12)	2 (15.4)
*Crocodylidea* (9)	15	0/0/15/0	n.a.	2 (13.3)
*Elapidea* (6)	11	11/0/0/0/	WB(11)	2 (18.2)
*Gekkonidae* (1)	1	0/0/1/0	n.a.	0
*Iguanidae* (1)	3	0/0/3/0	n.a.	3 (100)
*Pythonidae* (9)	23	0/14/4/5	WB(1)/CB(17)/n.a(5)	8 (34.8)
*Testudinidea* (3)	4	0/0/4/0	n.a.	1 (25.0)
*Varanidea* (6)	7	0/0/7/0	n.a.	0
*Viperidae* (5)	12	12/0/0/0	WB(7)/CB(4)/n.a.(1)	1 (8.3)

**Table 4:** Positive parasite test results in reptiles regarding infection with gastrointestinal endoparasites across a four investigated area. (total n = 571; 86 positive and 485 negative) (-: no detection of parasites; summary of result abbreviations in alphabetical order: ASC (Ascarid eggs); BAL (*Balantidium* cysts/trophozoites); CB (captive bred); CE (Cestodes); ENEM (eggs/larvae of free-living nematodes); HWE (Hookworm eggs); KAC (*Kalicephalus* spp.); KTE (*Kapsulotaenia* spp. egg); MYO (rodent specific fur mites, *Myocoptes*/*Myobia* spp., or their eggs); n.a. (not applicable); NYC (*Nyctotherus* cysts/trophozoites); OPA (*Ophidascaris* spp.); OXY (oxyurid worm); rtPCR results G (*Giardia* spp.), PS (pentastomes); S (*Strongyloide*s spp.), T (*Trichomonas* spp.), O (*Ophidascaris* spp.); STE (strongylid-type egg), STL (strongylid-type larvae.

Collection place	Species	Origin	Microscopy	rtPCR results				Dissection
				G	S	T	O	
Research herpetarium	*Bitis arietans*	CB	–	–	–	–	+	n.a.
	*Dendroaspis jamesoni*	Cameroon	STL	–	+	–	–	n.a.
	*Dendroaspis polylepis*	Tanzania	–	–	+	–	–	n.a.
	*Naja subfulva*	Cameroon	–	–	+	–	–	STL
	*Echis ocellatus*	Ghana	–	–	–	–	–	KAC
	*Echis ocellatus*	Ghana	–	–	–	–	–	KAC, CE, OXY
	*Echis ocellatus*	Ghana	–	–	–	–	–	PS
	*Bitis arietans*	n.a.	–	–	–	–	–	STE
Pet shop	*Boa constrictor*	CB	BAL, MYO	–	–	–	–	n.a.
	*Python regius*	CB	ASC, MYO	–	–	–	–	n.a.
	*Python regius*	CB	–	–	–	–	+	n.a.
	*Python regius*	CB	ASC	–	+	–	–	n.a.
	*Python regius*	CB	ASC	–	+	–	–	n.a.
	*Python regius*	CB	ASC	–	–	–	–	n.a.
	*Morelia spilota*	CB	ASC	–	–	–	–	n.a.
Zoos	*Stigmochelys pardalis*	n.a.	NYC	–	–	–	–	n.a.
	*Crocodylus moreletii*	n.a.	–	+	–	–	–	n.a.
	*Mecistops cataphractus*	n.a.	STE, ASC	–	–	–	–	n.a.
	*Furcifer pardalis*	n.a.	–	–	–	+	–	n.a.
	*Furcifer pardalis*	n.a.	–	–	+	–	–	n.a.
	*Brachylophus fasciatus*	n.a.	–	–	–	–	+	n.a.
	*Brachylophus fasciatus*	n.a.	STL	–	–	–	–	n.a.
	*Brachylophus fasciatus*	n.a.	–	–	–	–	+	n.a.
	*Morelia viridis*	n.a.	KTE, ENEM	–	–	–	–	n.a.
	*Morelia viridis*	n.a.	HWE, STL	+	–	–	–	n.a.
Pet animals	*Lampropeltis californiae*	CB	ASC, STL	–	–	–	+	n.a.
	*Elaphe carinata yonaguniensis*	CB Japan	–	–	–	–	+	n.a.

**Table 5:** Number and percentage of positive reptile regarding infection with gastrointestinal endoparasites (total n = 109; 27 positive and 82 negative).

Parasite species	No. of positive (%)	Host species (n)
Ascarids	Total: 10/109 (6.3)	*Colubridae*:
	*Colubridae* 2/13 (15.4)	*Lampropeltis californiae* (1), *Elaphe carinata yonaguniensis* (1)
	*Crocodylidae* 1/15 (6.7)	*Crocodylidae*: *Mecistops cataphractus* (1)
	*Iguanidae* 1/3 (33.3)	*Iguanidae*: *Brachylophus fasciatus* (1)
	*Pythonidae* 5/23 (21.7)	*Pythonidae*: *Python regius* (4), *Morelia viridis* (1)
	*Viperidae* 1/12 (8.3)	*Viperidae*: *Bitis arietans* (1),
*Balantidium* spp.	Total: 1/109 (0.9)	*Boidae*: *Boa constrictor* (1)
	*Boidae* 1/3 (33.3)	
Cestodes	Total: 1/109 (0.9)	*Viperidae*: *Echis ocellatus* (1)
	*Viperidae* 1/12 (8.3)	
*Giardia* spp.	Total: 2/109 (1.8)	*Crocodylidae*: *Crocodylus moreletii* (1)
	*Crocodylidae* 1/15 (6.7)	*Pythonidae*: *Morelia viridis* (1)
	*Pythonidae* 1/23 (4.4)	
Hookworms	Total: 3/109 (2.8)	*Pythonidae*: *Morelia viridis* (1)
	*Pythonidae* 1/23 (4.4)	*Viperidae*: *Echis ocellatus* (2)
	*Viperidae* 2/12 (16.7)	
*Kapsulotaenia* spp.	Total: 1/109 (0.9)	*Pythonidae*: *Morelia viridis* (1)
	*Pythonidae* 1/23 (4.4)	
*Nyctotherus* spp.	Total: 1/109 (0.9)	*Testudinidea*: *Stigmochelys pardalis* (1)
	*Testudinidea* 1/4 (25)	
Pentastomes	Total: 1/109 (0.9)	*Viperidae*: *Echis ocellatus* (1)
	*Viperidae* 1/12 (8.3)	
*Strongyloides* spp.	Total: 6/109 (5.5)	*Chamaeleonidae*: *Furcifer pardalis* (1)
	*Chamaeleonidae* 1/3 (33.3)	*Elapidae*: *Dendroaspis jamesoni* (1), *Dendroaspis polylepis* (1), *Naja subfulva* (1)
	*Elapidea* 3/11 (27.3)	*Pythonidae*: *Python regius* (2)
	*Pythonidae* 2/23 (8.7)	
*Trichomonas* spp.	Total: 1/109 (0.9)	*Chamaeleonidae: Furcifer pardalis* (1)
	*Chamaeleonidae* 1/3 (33.3)	

**Fig. 1:**
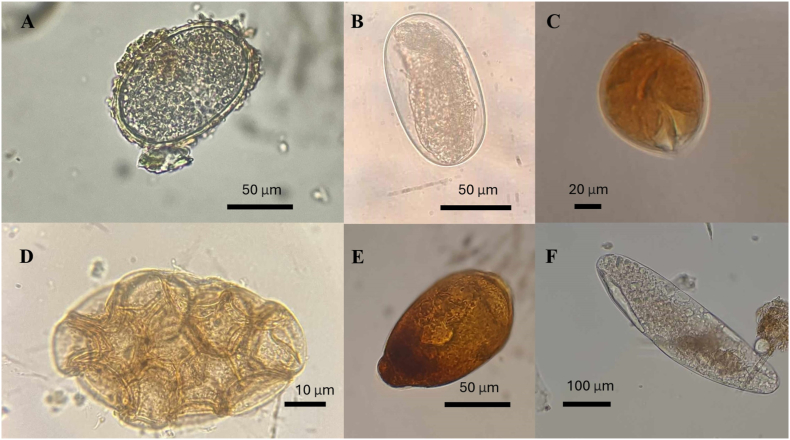
Different parasite stages in reptile faecal samples identified by sedimentation-flotation techniques: A) Ascarid egg B) a typical hookworm egg with a thin egg-shell wall containing a morula inside C) Trophozoite of *Balantidium* spp., D) Ciliate cyst (*Nyctotherus* spp.) with iodine staining, E) Characteristic egg cluster (*Kapsulotaenia* spp.) F) Mite egg (*Myocoptes musculinus* like).

Microscopy identified parasitic eggs, cysts, and larvae in several species, with notable detections including ascarid eggs in *Python regius* and *Morelia spilota* from pet shops (Fig. 1A), strongylid type eggs and larvae in *Dendroaspis jamesoni* and *Morelia viridis* (Fig. 1B), and *Balantidium* cysts in *Boa constrictor* (Fig. 1C). Additionally, *Nyctotherus cysts* were detected in *Stigmochelys pardalis* (Fig. 1D). The cestodes identified by their egg cluster (*Kapsulotaenia* spp.) from *Morelia viridis* (Fig. 1E). One case of co-infection was found in a Slender snouted crocodile (*Mecistops cataphractus*) harbouring both *Ascarid* and *Strongylid* type eggs. Due to reptiles predatory behaviour, endo- and ectoparasites from various prey species can appear as transient pseudoparasites within their intestinal tract. For example, mite eggs (*Myocoptes/Myobia* spp.) specific to rodents were observed as pseudoparasitic ‘infections’ in seven reptile samples, most commonly in pet shop-sourced reptiles (Fig. 1F–Table 3., Online supplement).

The rtPCR analysis improved detection sensitivity, identifying additional protist and nematode infections missed by microscopy. *Giardia* spp. was detected in *Furcifer pardalis* from zoo collections, while *Strongyloides* spp. were identified in *Dendroaspis polylepis*. Additionally, *Trichomonas* spp. was detected in *Furcifer pardalis* and *Ophidascaris* spp. in more species (Table 4, Table 5), with *Ophidascaris* prevalence spanning both captive-bred and wild-caught hosts. The rtPCR analysis detected *Ophidascaris* spp., in six of 109 samples (5.5%), spanning a range of reptile hosts, including *Bitis arietans*, *Python regius*, and *Lampropeltis californiae*. All rtPCR target organisms were found in 11 species. *Strongyloides* infection in *Dendroaspis jamesoni* showed a lower cycle threshold (Ct) value of 24.13, compared to a median Ct of 31.36 for rtPCR-only positives, underscoring the increased sensitivity of molecular methods.

**Fig. 2:**
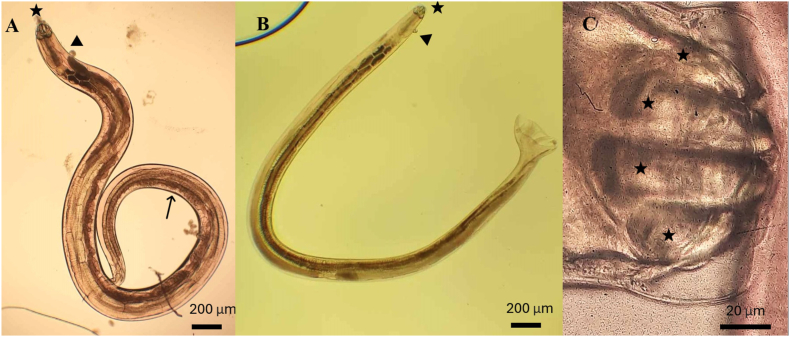
Microscopic image of a A) gravid female, B) male *Kalicephalus* spp. observed in the stomach of *Echis ocellatus*. The adult worms' measures approximately 7 mm in length, featuring a distinctive kalicephal-type capsule (indicated by a star), a short, robust esophagus with a prominent round esophageal bulb (arrowhead), numerous eggs within the uterus (long arrow), and a terminal spike at the tail end (short arrow). C) Characteristic head end of ancylostomatid *Kalicephalus* spp., the prominent kalicephal-type capsule consists of four anterior plates (stars).

**Fig. 3:**
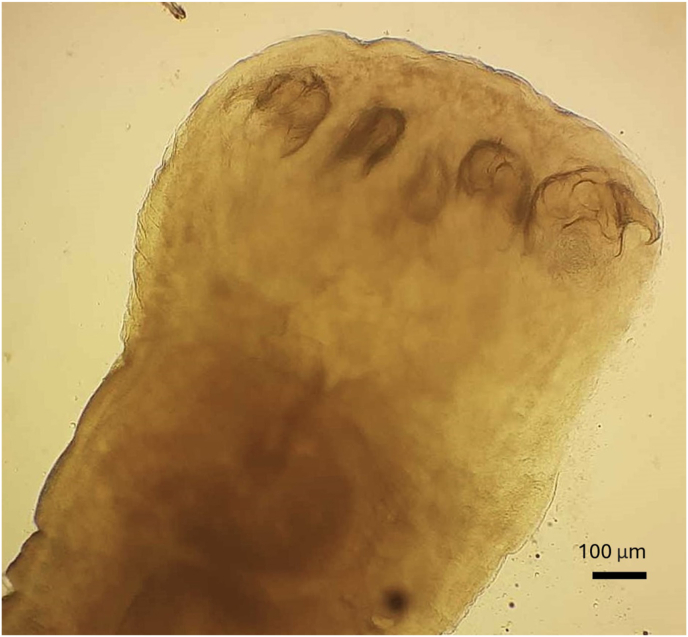
Photograph of immature pentastomes, showing with anterior hooks.

**Fig. 4:**
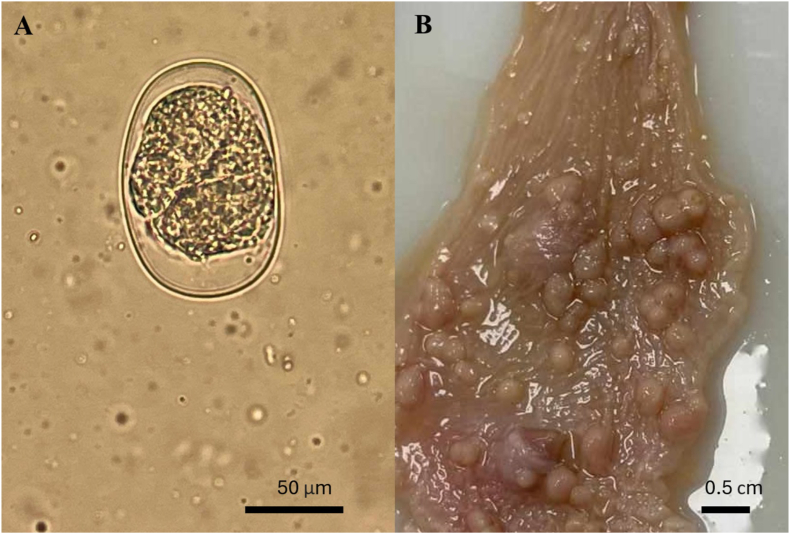
A) *Strongyloides* egg found in B) ulcer-like structures of *Bitis arietans* stomach.

Dissection confirmed and revealed additional parasitic infections that were undetectable via non-invasive methods (Table 1, Table 4). Among the snakes inspected by necropsy, *Kalicephalus* spp. was the most frequently observed parasite (Fig. 2) in herpetarium collections, 20 female and 14 male worms were detected in *Echis ocellatus*. The examined snake died one week after its arrival from Ghana. Two immature pentastomes, a zoonotic parasite, were identified on the lung surface in another specimen of *Echis ocellatu*s (Fig. 3) (Vasaruchapong et al., 2017). Furthermore, *Strongyloides* eggs were detected within ulcer-like lesions on the stomach walls of one *Bitis arietans* (Fig. 4A and B). Notably, 55.6% of the nine necropsied animals harboured latent parasitic infections.

No significant differences were observed in the overall prevalence of parasitic infections between CB and WC reptiles (p > 0.05). Wild-caught reptiles showed higher nematode diversity, notably Strongylid species, whereas captive-bred reptiles exhibited a greater prevalence of protist infections, such as *Giardia*. Of note *Giardia* spp. was present in two species by rtPCR. *Balantidium* spp. and *Trichomonas* spp. were also detected (Table 4, Table 5).

Coinfections with multiple parasite groups were identified in 22.2% of infected reptiles. The combination of these techniques provided the most comprehensive parasitic identification.

## 4 Discussion

This pilot study provides valuable insights into the prevalence and diversity of endoparasites in CB and WC exotic reptiles in the UK, emphasising the necessity of employing a combination of diagnostic techniques for accurate parasitic detection. The observed prevalence of parasitic infection (21.1%) is consistent with previous findings, including a 21.5% infection rate in snakes imported to Slovakia (Halan and Kottferova, 2021), 44.6% from exotic pet lizards in Texas, United States (Ellerd et al., 2022) and 47.3% in snakes imported to Slovenia (Rataj et al., 2011). Okulewicz et al. (2014) reported helminth infections in 13.7% of 28 captive snakes in the City Zoological Garden of Wrocław and a zoological wholesale facility.

Rhabditoidean helminths, particularly *Strongyloides*, are recognised as significant nematodes (Frank, 1981), in that species of *Strongyloides* are known to frequently infect wild snakes as well as those kept in captivity. Members of the family *Boidae* are particularly susceptible to intestinal *Strongyloides* species (Pasmans et al., 2008; Beck and Pantchev, 2013). In Hallinger et al. (2020), a total of 2.6% of the snakes tested positive for *Rhabdias/Strongyloides*. In our study, we found that 5.6% of the reptiles tested positive for *Strongyloides* via rtPCR. Strongyloidosis can lead to clinically manifested enteritis in reptiles, making it an important differential diagnosis in cases of intestinal disorders (Beck and Pantchev, 2013).

The thick-walled eggs were assumed to belong to ascaridoid nematodes, which are frequently found in pythonid and colubrid snakes. Ascarid nematodes are one of the most important pathogens of snakes, and infestation can be fatal (Beck and Pantchev, 2013). A zoonotic case of human neural larva migrans caused by the ascarid nematode, *Ophidascaris robertsi*, diagnosed after removing a nematode from the brain of a woman with hypereosinophilic syndrome (Hossain et al., 2023).

*Strongyloides* were more common in WC reptiles, while protozoa, such as *Giardia*, were more prevalent in CB snakes. These findings align with previous research suggesting that WC reptiles face greater exposure to parasites in their natural habitats due to intricate ecological interactions, while CB reptiles may be more vulnerable to protist infections stemming from suboptimal hygiene and biosecurity practices in captivity (Rataj et al., 2011; Fernandes et al., 2004; Ellerd et al., 2022).

While microscopy is effective for identifying morphologically distinct parasites, it lacks sensitivity for low-intensity infections or intermittent shedders, such as *Giardia* and *Trichomonas* (Ndao, 2009; Hallinger et al., 2019). In this study, rtPCR detected several infections missed by microscopy, confirming its high sensitivity and specificity, particularly for protist and nematode parasites (Verweij et al., 2007; Jothikumar et al., 2021). The ciliated protist *Balantidium* spp. and *Nyctotherus* spp. identified have been shown to be important commensal organisms, but may reach high levels in the presence of gastrointestinal diseases (Girling et al., 2004). Sedimentation and flotation microscopy was effective for identifying morphologically distinct parasites but missed low-intensity infections, such as *Giardia* spp. and *Trichomonas* spp. (Hoffman et al., 1934).

Necropsy played a crucial role in detecting latent infections, particularly cestodes, where eggs were not visible in faecal samples. This highlights the significance of post-mortem examinations, especially in cases where parasites localize in tissues or organs, or when the host has subclinical or pre-patent infection. Furthermore, necropsy facilitated the identification by adult *Kalicephalus* worms and their eggs (Jacobson and Garner, 2020). Infection with *Kalicephalus* causes mild enteritis in snakes leading to secondary bacterial infection and sometimes death (Matt et al., 2020.). One limitation was the relatively small sample size for necropsies, which may not fully represent latent infections across species.

It is crucial that every reptile kept in captivity undergoes post-mortem parasitological examination, regardless of the cause of death or the existence of a parasitological programme. Such systematic examinations would provide accurate data on the prevalence of parasites and their fluctuations over time. While random investigations may yield interesting findings, they often lack the necessary consistency to draw meaningful conclusions. In the case of latent infections, regular interim screening in captive populations could help identify infections that are otherwise undetected. Prepatent infections, which eventually become patent, are only discovered through frequent monitoring. While quarantine protocols may be less expensive than regular examinations, parasitological testing should always be a part of the quarantine process. Furthermore, understanding which parasites are commonly present in a given population could enable the use of targeted PCR assays, making the process more efficient, especially in larger sample sizes and frequent screenings. From a pathological standpoint, the focus should be on those parasites that are most prevalent in the population. As this was a pilot study, species-level identification of parasites was not included within the scope of the current objectives. However, the importance of such detailed analyses is recognised, and their inclusion is planned for future studies.

These findings carry substantial implications for reptile management in both conservation and captivity. For WC reptiles, quarantine measures and routine parasitic screening upon entry into captivity can help prevent the introduction and spread of parasites among CB populations. Conversely, for CB reptiles, enhancing hygiene and biosecurity protocols, especially in breeding facilities and pet trade environments, is crucial for mitigating infections (Pienaar et al., 2022).

The zoonotic potential of some parasites identified in this study, such as *Giardia*, *Ophidascaris* and pentastomes raises public health concerns. Close contact between humans and reptiles, particularly in the exotic pet trade or research settings, can facilitate the transmission of zoonotic pathogens (Hossain et al., 2023; Ioannou and Vamvoukaki, 2019). Thus, adopting a One Health approach that integrates veterinary, public health, and environmental disciplines is essential for controlling zoonotic parasites and ensuring the health and welfare of both reptiles and humans (Cunningham et al., 2017).

## 5 Conclusion

This study demonstrates the value of combining multiple diagnostic techniques for assessing parasitic infections in exotic reptiles. The findings contribute to our understanding of parasite transmission in both CB and WC reptiles and offer important insights for improving captive management practices, reducing zoonotic risks, and safeguarding reptile welfare. Future research should focus on expanding parasitic screening across a broader range of reptile species and exploring the efficacy of integrated parasite management strategies in different environments.

## CRediT authorship contribution statement

**Shea Murray:** Writing – review & editing, Writing – original draft, Formal analysis, Data curation. **Lucas J. Cunningham:** Writing – review & editing, Validation, Supervision, Methodology, Investigation. **Paul Rowley:** Writing – review & editing, Investigation, Formal analysis, Data curation. **Edouard Crittenden:** Writing – review & editing, Formal analysis, Data curation. **Nicholas R. Casewell:** Writing – review & editing, Formal analysis, Data curation. **E. James LaCourse:** Writing – review & editing, Project administration, Conceptualization. **J. Russell Stothard:** Writing – review & editing, Visualization, Supervision, Data curation, Conceptualization. **Alexandra Juhász:** Writing – review & editing, Writing – original draft, Visualization, Validation, Supervision, Methodology, Investigation, Formal analysis, Data curation, Conceptualization.

## Ethical approval

The authors assert that all procedures contributing to this work comply with the ethical standards of the relevant national and institutional guides on the care and use of vertebrates. Ethical approval for the study was granted by the Liverpool School of Tropical Medicine's Ethics Committee (Ref. No. LSTM/2023/ETH007). No experiments were performed on animals.
